# Epitope imprinted polymers: a versatile and cost-effective alternative for targeted therapies

**DOI:** 10.17179/excli2025-8306

**Published:** 2025-05-26

**Authors:** Liming Zhang, Md Sadique Hussain, Mudasir Maqbool, Sumel Ashique, Gyas Khan

**Affiliations:** 1 School of Basic Medical Sciences, Tsinghua University, Beijing 100084, China; 2 Uttaranchal Institute of Pharmaceutical Sciences, Uttaranchal University, Dehradun, Uttarakhand 248007, India; 3 Department of Pharmaceutical Sciences, University of Kashmir, Hazratbal, Srinagar 190006, Jammu and Kashmir, India; 4 School of Pharmaceutical Sciences, Lovely Professional University, Phagwara, Punjab 144411, India; 5 Department of Pharmacology and Toxicology, College of Pharmacy, Jazan University, Jazan, Saudi Arabia

## ⁯⁯

Natural receptors, including antibodies, play a crucial role in targeted medication administration and immunotherapy due to their exceptional sensitivity and efficacy. However, their limitations, such as intricate manufacturing methods, high costs, and susceptibility to harsh environments, pose obstacles to their widespread application (Xing et al., 2022[7]). Epitope imprinted polymers (EIPs) are emerging as a revolutionary alternative to natural receptors. These synthetic receptors, which can be target proteins, peptides, and small compounds with high precision, provide greater reliability, flexibility, and adaptability. Their resistance against breakdown and nonspecific binding makes them ideal for drug delivery, diagnostic uses, and disease-specific surveillance (Xu et al., 2019[8], Zouani et al., 2013[9]).

Recent advances underscore the promise of EIPs in immunotherapy and oncological care. Lu et al. demonstrated the effectiveness of molecularly imprinted lysosomal nanodegraders (MILND) in obstructing the PD-L1/PD-1 axis in breast cancer, achieving a suppression rate of 74.2% in a 4T1 model via the degradation of intracellular PD-1 (Lu et al., 2024[3]). Similarly, structural modification of epitope motifs have become an essential component. Qin et al. (2019[6]) showed that EIPs with α-helical frameworks not only improved binding affinity but also increased phagocytic activity of immune cells against cancer cells, emphasizing the therapeutic value of 3D structural insights.

Furthermore, the application of EIPs in immunity modulation presents promising opportunities for less invasive cancer treatments. Engineered nanoparticles targeting triple-negative breast cancer (TNBC) exhibited enhanced efficiency by recruiting natural killer (NK) cells and macrophages, hence increasing antibody-mediated cell death and phagocytosis to 71.6% (Guan et al., 2023[1]). These findings suggest that EIPs could revolutionize cancer therapy by reducing the adverse effects of conventional treatments and enabling precision medicine-based interventions.

Looking ahead, EIPs are set to transform personalized healthcare via the integration of advanced technologies. These high-affinity polymers were initially characterized by their binding to epitope regions by solution saturation transfer difference and gradient-enhanced NMR spectroscopy (Mier et al., 2021[4]). Herrera León et al. (2023[2]) also noted changes in STDs-NMR signal peaks. Additionally, novel therapeutic strategies combining chemotherapy with photodynamic therapy (PDT) have been proposed (Peng et al., 2020[5]). Computational modeling and artificial intelligence (AI) are expected to further optimize EIP design, facilitating the development of highly specific and robust EIPs for a wide range of diseases. Their combination with emerging modalities, such as gene therapy and CRISPR technology, may significantly improve therapeutic outcomes. Moreover, their integration with external triggers like photodynamic or magnetically induced therapies holds potential for synergistic, multimodal therapeutic approaches.

From a clinical perspective, the large-scale production of EIPs must be refined to meet regulatory standards, ensure repeatability, and lower production costs. Establishing standardized protocols for evaluating their biocompatibility and long-term efficacy will be essential for their clinical adoption. Furthermore, investigating their function in modulating intracellular mechanisms and leveraging their potential in theranostics could open new frontiers in personalized medicine.

### Conclusion

EIPs represent a transformative advancement in targeted therapies, offering enhanced stability, cost-effectiveness, and remarkable adaptability. As research progresses, their incorporation into complex therapeutic systems is expected to overcome current limitations in treatment modalities, paving the way for innovative and personalized medical solutions.

## Declaration

### Acknowledgments

None.

### Author contributions

Liming Zhang: Data curation, Writing - original draft. Md Sadique Hussain: Conceptualization, Data curation, Writing - original draft. Gyas Khan: Formal analysis, Writing - review & editing. Sumel Ashique: Conceptualization, Investigation. Mudasir Maqbool: Methodology, Writing - original draft.

All authors have approved the final version of the manuscript.

### Funding

None.

### Data Availability

Not Applicable.

### Conflict of interest

None to declare.

### Consent for Publication

Not Applicable.

### Declaration of Generative AI and AI-assisted technologies in the writing process

During the preparation of this work, the author(s) used ChatGPT to correct the grammatical and typographical errors in the manuscript. All authors have approved the final version of the manuscript.
